# The Anaphylaxis of Poor Air

**Published:** 1911-04

**Authors:** 


### The Anaphylaxis of Poor Air

THE modern and more accurate and intelligent consideration of the cause of disease must ever be the dual concept of the soil and of the seed. As in nature so in pathogenesis, the soil is much but not all, the seed is much but not everything. What we know, and what we may know of anaphylaxis is of enormous importance, practical and theoretical.

It may widen our horizon distinctly to recall that we may have a specific anaphylaxis for each communicable disease. This anaphylaxis may control the incidence, the course that it shall be long, short or medium, mild, severe, malignant, and may decide the termination that it shall be in recovery complete or partial, or by permanent local disability, or death. The causes of anaphylaxis include many of the more important elements of a man's heredity and environment—the air we breathe, the water we drink, the other foods we use, the clothes we wear or should wear, our habits of rest and unrest, of exercise and recreation, what our fathers did or left undone, are some of the causes of anaphylaxis.

The practical importance of anaphylaxis should come to our minds when we recall the fact that to anaphylaxis we owe most, if not all, of our cases of tuberculosis, of pneumonia, of bronchitis, of influenza, of colds, of acute and chronic catarrh, of scarlet fever, of diphtheria, of so-called poliomyelitis, in fact of every disease the infection of which may be air borne and which may enter the system through any portion of the respiratory tract. We might also do well to remember that anaphylaxis may be local or general, involving a part only as the mucosa of the nasopharynx, or including the entire system; or that the anaphylaxis may be only for some specific infection and not for others. Our laboratory men know that the infection of tuberculosis may be present in the nose of a child for months and years without harm, and believe that in like manner the infection of the so-called poliomyelitis may be lodged in safety, that in both these cases and many similar infections, a local anaphylaxis caused possibly by the child or person breathing the carbon monoxide poisoned air of a room recently occupied by an indoor tobacco burner, determines and causes systemic invasion of the infection, the subsequent death or lifelong paralytic affliction of the survivor. When the complete rôle of the local anaphylaxis of carbon monoxide and the nicotins shall be written it will constitute a chapter of etiology of enormous significance.

Our main purpose, however, is to invite attention to the rôle in the production of general anaphylaxis of the air we breathe. Here again we have a distinct consensus of the art of medicine—the observations of practical men, and the findings of the workers in the laboratories.

All the way from the Black Hole of Calcutta through the countless private homes where foul air is the rule and fresh air the rare exception, through the many offices and workrooms of our factories, where the heating is by pipeless stoves, to the class-rooms of our unventilated public schools, where, at the command of the law, thousands of our sensitive children are obliged to spend many hours of their time, through the entire circuit crawls, or hides the slimy serpent, the enemy of man—lack of pure air to breathe, necessity of drawing with each respiration the full of the lungs of air befouled with respiratory excrement and carbonic acid in large excess, a condition which neither animals nor man can endure for any length of time without causing general anaphylaxis.

We may safely conclude that the incidence of tuberculosis, of pneumonia, of influenza, of colds, of scarlet fever, of diphtheria, of whooping cough, and of so-called poliomyelitis would be diminished well nigh to extermination if we might all, in our work and in our play, enjoy the blessing of pure air—be delivered from the anaphylaxis of nonventilation.

---

Sir James Crichton Browne, of London (*The Tribune*) described recently, at the end of a dinner, how a delicate morsel, perfectly served, of delicious flavor and good aroma, will send to the stomach, before it is swallowed, a telephone message to say that it is coming.

Such a morsel, he said, not only sets the mouth watering by stimulating the salivary glands, but it also induces a flow of the gastric juices by acting on the glands of the stomach. These glands it brings into play before any portion is swallowed. It is, in fact, telephoning down to the stomach to say that something good is coming, and the stomach immediately prepares itself for its reception.

A nasty or insipid dish has no such effect. If it is nasty the stomach rejects it; if insipid, it receives it with comparative indifference. It is of the utmost importance, he held, that good flavor and good aroma should prevail, for nice food is more easily assimilated than that which is flavorless, and good cooking not merely tickles the palate, but it also contributes to the great work of nutrition.

The JOURNAL announces to its many patrons that it will continue to be published upon the same lines as heretofore, with certain improvements, as directed in Dr. Potter's will.

Reviews of books not appearing in this number of the JOURNAL will be published in the May issue.

